# Targeted radiotherapy of pigmented melanoma with ^131^I-5-IPN

**DOI:** 10.1186/s13046-018-0983-0

**Published:** 2018-12-11

**Authors:** Xiaodong Xu, Lujie Yuan, Yongkang Gai, Qingyao Liu, Lianglan Yin, Yaqun Jiang, Yichun Wang, Yongxue Zhang, Xiaoli Lan

**Affiliations:** 1 Department of Nuclear Medicine, Wuhan Union Hospital, Tongji Medical College, Huazhong University of Science and Technology, No. 1277 Jiefang Ave, Wuhan, 430022 Hubei Province China; 2 Hubei Key Laboratory of Molecular Imaging, Wuhan, 430022 China

**Keywords:** Melanoma, Melanin, Targeted radionuclide therapy, Picolinamide

## Abstract

**Purpose:**

There has been no satisfactory treatment for advanced melanoma until now. Targeted radionuclide therapy (TRNT) may be a promising option for this heretofore lethal disease. Our goal in this study was to synthesize ^131^I-N-(2-(diethylamino)ethyl)-5-(iodo-131I)picolinamide (^131^I-5-IPN) and evaluate its therapeutic ability and toxicity as a radioiodinated melanin-targeting therapeutic agent.

**Methods:**

The trimethylstannyl precursor was synthesized and labeled with ^131^I to obtain ^131^I-5-IPN. The pharmacokinetics of ^131^I-5-IPN was evaluated through SPECT imaging, and its biodistribution was assessed in B16F10 tumor models and in A375 human-to-mouse xenografts. For TRNT, B16F10 melanoma-bearing mice were randomly allocated to receive one of five treatments (*n* = 10 per group): group A (the control group) received 0.1 mL saline; group B was treated with an equimolar dose of unlabeled precursor; group C received 18.5 MBq of [^131^I]NaI; group D and E received one or two dose of 18.5 MBq ^131^I-5-IPN, respectively. TRNT efficacy was evaluated through tumor volume measurement and biology study. The toxic effects of ^131^I-5-IPN on vital organs were assessed with laboratory tests and histopathological examination. The radiation absorbed dose to vital organs was estimated based on biodistribution data.

**Results:**

^131^I-5-IPN was successfully prepared with a good radiochemistry yield (55% ± 5%, *n* = 5), and it exhibited a high uptake ratio in melanin-positive B16F10 cells which indicating high specificity. SPECT imaging and biodistribution of ^131^I-5-IPN showed lasting high tumor uptake in pigmented B16F10 models for 72 h. TRNT with ^131^I-5-IPN led to a significant anti-tumor effect and Groups D and E displayed an extended median survival compared to groups A, B, and C. The highest absorbed dose to a vital organ was 0.25 mSv/MBq to the liver; no obvious injury to the liver or kidneys was observed during treatment. ^131^I-5-IPN treatment was associated with reduction of expression of proliferating cell nuclear antigen (PCNA) and Ki67 and cell cycle blockage in G2/M phase in tumor tissues. Decreased vascular endothelial growth factor and CD31 expression, implying reduced tumor growth, was noted after TRNT.

**Conclusion:**

We successfully synthesized ^131^I-5-IPN, which presents long-time retention in melanotic melanoma. TRNT with ^131^I-5-IPN has the potential to be a safe and effective strategy for management of pigmented melanoma.

## Introduction

An estimated 132,000 new cases of melanoma are diagnosed every year worldwide [1]. Patients with distant metastases still have a poor prognosis, the 5-year survival is only 17% [2]. The common first-line therapy such as dacarbazine with a complete response of approximately 5% is of very limited benefit for advanced melanoma [3]. In recent years, our knowledge of the molecular biology and immunoregulatory mechanisms in melanoma has greatly expanded, which has brought about great advances for treatment of this lethal cancer. Several novel targeted therapeutic agents including immunotherapy drugs have been developed and approved for advanced melanoma treatment [2], including BRAF inhibitors (vemurafenib), anti-CTLA-4 agents (ipilimumab), and anti-PD-1 agents (nivolumab and pembrolizumab). While a portion of patients respond well to these therapies in clinical trials, adaptive resistance, serious adverse effects, and high cost present challenges for their further application. In addition, more evidence on long-term benefit still needs to be collected. In this context, radiotherapy and targeted radionuclide therapy (TRNT) of melanoma has recently been receiving attention [4, 5].

In clinical practice, TRNT has achieved great success against radiosensitive tumors, particularly neuroendocrine tumors [6] and lymphomas [7, 8]. For melanoma, several different carrier molecules are currently being labeled with radionuclides for TRNT, such as ^111^In labeled monoclonal antibody (mAb) KM871 targeting GD3 expressed on the surface of melanoma cells [9], ^188^Re-labled mAb 6D2 targeting intracellular melanin antigen [10], and more and more peptides used for both radioimmunotherapy [11–13] and detection [14] of melanoma. During the past decade, many small benzamide molecules radiolabeled with ^18^F [15–17], ^11^C [18], or ^131^I [19–21] have been developed for diagnosis or therapy of melanoma. More recently, we reported two promising ^18^F-fluoro radiotracers containing ^18^F-5-fluoro-N-(2-(diethylamino)ethyl)picolinamide (^18^F-5-FPN) [22] and its optimized version (^18^F-PEG_3_-FPN) [23], for pigmented melanoma positron-emission tomography (PET) detection. High specificity and affinity as well as rapid elimination from nontarget organs make them potential carriers for TRNT. To verify this hypothesis, we prepared ^131^I-N-(2-(diethylamino)ethyl)-5-(iodo-131I)picolinamide (^131^I-5-IPN) through a radioiododestannylation reaction based on a trimethylstannyl precursor, and the in vivo distribution and the anti-tumor ability of ^131^I-5-IPN were investigated in pigmented B16F10 tumor models. The molecular events of ^131^I-5-IPN slowing down the growth of tumor were recorded with western blotting, immunofluorescence, and flow cytometry. We also analyzed the adverse effects after TRNT with ^131^I-5-IPN. The main aim of this study was to evaluate the efficacy and to further characterize adverse effects of ^131^I-5-IPN in vivo in melanoma treatment.

## Materials and methods

### Reagents and instruments

Sodium iodide [^131^I]NaI oral solution was provided by HTA Co., Ltd. Other chemicals and solvents were obtained from the following companies: Acros Organics (USA), J&K Chemicals (Beijing, China) and Sigma-Aldrich (St. Louis, MO, USA). Thin layer chromatography was performed on silica gel thin layer chromatography plates (Anhui LiangChen Silicon Material Co. Ltd., China) visualized under UV light (254 nm). Compounds were identified with a NMR spectrometer (NMR spectra Bruker 400 MHz, Bruker, Germany) and an ion-trap mass spectrometer (Thermo LCQ DECA XP^plus^ ESI-MS, Thermo Fisher, USA). Radiochemical purity was analyzed by analytic high-pressure liquid chromatography (HPLC) with a flow count radiation detector (Meinaite, Germany) using a column (Elite Hypersil® ODS/ODS2 C-18 column, Alltech, USA) (4.6 × 250 mm, 5 μm particle size). The mobile phase started from acetonitrile (MeCN)/water (5,95, *v*/v) containing 0.1% trifluoroacetic acid (TFA) at 0–3 min, then ramped to 90:10 at 25 min at a flow rate of 1.0 mL/min.

### Synthesis of N-(2-(diethylamino)ethyl)-5-iodopicolinamide (1)

A solution of 5-iodopicolinic acid (100 mg, 0.40 mmol) and 20 μL (a catalytic amount) of *N,N*-dimethyl formamide in thionyl chloride was refluxed for 5 h at 60 °C under a nitrogen atmosphere. The excess thionyl chloride was removed under reduced pressure, and crude 5-iodine-2-pyridinecarbonyl chloride was obtained. Subsequently, *N,N*-diethylenediamine (0.48 mmol, 332.68 mg), K_2_CO_3_ (100 mg) and 5 mL of dichloromethane (DCM) were added to a three-neck flask, and the flask was cooled to 0 °C in an ice bath. 5-iodine-2-pyridinecarbonyl chloride dissolved in 5 mL of dry DCM was added dropwise over 10 min. We then allowed the solution to warm to room temperature and stirred the mixture overnight. We washed the mixture with water (3 × 10 mL) and re-extracted the aqueous fraction with DCM. The combined organic layers were dried over anhydrous Na_2_SO_4_. The mixture was filtered and concentrated under reduced pressure to obtain crude material. The crude mixture was purified by silica gel chromatography (DCM/methanol [MeOH] 20:1) to afford compound 1 (121 mg, 73% yield) as a tawny oil. ^1^H NMR (400 MHz, CDCl_3_) δ 8.75 (s, 1H), 8.28 (s, 1H), 8.13 (d, J = 10.1 Hz, 1H), 7.93 (d, J = 8.2 Hz, 1H), 3.48 (q, J = 6.0 Hz, 2H), 2.78–2.41 (m, 6H), 1.02 (t, J = 7.1 Hz, 6H).^13^C NMR (101 MHz, CDCl_3_) δ 162.78, 153.23, 147.93, 144.58, 122.82, 95.76, 50.48, 46.06, 36.23, 10.87. HRMS m/z (ESI+): calcd for [M + H]^+^ 348.0495, found 348.0551.

### Preparation of N-(2-(diethylamino)ethyl)-5-(trimethylstannyl)picolinamide (2)

A solution of compound **1** (10 mg, 0.08 mmol), hexamethylditin (34.4 mg, 0.21 mmol) and *N,N*-Diisopropylethylamine (1.2 eq) in MeCN (1.5 mL, 0.1 M) was added to a quartz test tube containing a small magnetic stirring bar, then the mixture was deoxygenated with argon for 10 min. The reaction mixture was irradiated using a 300 W high-pressure mercury lamp for 2 h [24]. The crude mixture was concentrated and purified by silica gel column chromatography, eluting with MeOH/DCM (1:10), to produce compound **2** (Trimethylstannyl precursor) (4.0 mg, 40%) as a colorless oil. ^1^H NMR (400 MHz, CDCl_3_) δ 8.66 (s, 1H), 8.60 (s, 1H), 8.03 (d, J = 7.5 Hz, 1H), 7.92 (d, J = 7.6 Hz, 1H), 3.90 (d, J = 4.9 Hz, 2H), 3.17 (s, 2H), 3.07 (s, 4H), 1.35 (t, J = 7.0 Hz, 6H), 1.23 (s, 9H). HRMS m/z (ESI^+^): calcd for [M + H]^+^ 386.1254, found 386.1247.

### Preparation of N-(2-(diethylamino)ethyl)-5-(iodo-^131^I)picolinamide (^131^I-5-IPN)

[^131^I] NaI (40 mCi), chloramine-T (20 μL, 1 mg/mL in water), and an aqueous HCl solution (50 μL, 1 M) was added to a solution of compound **2** (50 μL,1 mg/mL in ethanol) in a 1.5 mL Eppendorf tube. After reaction at 37 °C for 1 h, the solution was quenched with a 1 N aqueous NaOH solution (100 μL) and an aqueous sodium metabisulfite solution (10 μL, 20 mg/mL). The reaction mixture was purified though a kieselguhr (Extrelut, Sigma-Aldrich) column with water (20 mL) and ethanol (2 × 0.5 mL). The resulting radioiodinated compound (^131^I-5-IPN) was first dried under nitrogen, and then redissolved in saline and filtered using a 0.22 μm aseptic membrane filter for subsequent studies.

### Stability of ^131^I-5-IPN in human serum

As in our previous method [23],^131^I-5-IPN (74 kBq, 20 μCi) and human serum (0.2 mL) were incubated at 37 °C for 24 h. The radiochemical purity of ^131^I-5-IPN was identified by analytic HPLC.

### Octanol-water partition coefficient (log *P*)

Approximately 74 kBq (20 μCi) of ^131^I-5-IPN, 1.0 mL of phosphate-buffered saline (PBS), and 1.0 mL of octanol was added to a microcentrifuge tube. The mixture was vigorously vortexed (3000 rpm, 5 min) at room temperature. After centrifugation (13,000 rpm, 5 min), 3 aliquots (100 μL) of each layer were sampled, then the radioactivity was recorded with a γ-counter (2470 WIZARD; PerkinElmer, USA). The partition coefficient was calculated as the following formula: Log *P* = Log10 (counts in 1-octanol/counts in PBS).

### In vitro cell assay

B16F10 (murine pigmented melanoma) and A375 (human amelanotic melanoma) cells were purchased from a cell bank (Shanghai, China). Both cell lines were cultured in Dulbecco’s modified Eagle’s medium (DMEM) (Gibco, USA) containing 10% fetal bovine serum (Gibco) and kept in an incubator with 5% CO_2_ at 37 °C.

For the cell uptake assay, melanin-positive B16F10 and amelanotic A375 cells were plated in 24-well plates at a density of 1 × 10^5^ cells/well and incubated for 24 h. Before the experiment, cells were washed three times with cold PBS, and cultured with DMEM without fetal bovine serum for 1 h. Next, cells were incubated for 1, 3, and 6 h with 100 μL ^131^I-5-IPN (2 μCi, 1.23 pmol) at 37 °C. At a designated time point, the medium was removed and the cells were washed with ice-cold PBS and lysed with 1 N NaOH at room temperature for 5 min. The combined washes and lysate were measure with a γ-counter. Similarly, in the blocking experiment, the cells were pretreated using 1000-fold non-radioactive I-5-IPN (1.23 nmol, 0.43 μg) 30 min before adding ^131^I-5-IPN. After incubating at 37 °C for 1 h, the medium was removed and washed with ice-cold PBS and lysed with 1 N NaOH at room temperature for 5 min.

### Tumor model

All experimental schemes were approved by the Institutional Animal Care and Use Committee of Tongji Medical College of Huazhong University of Science and Technology. Five-week-old male C57BL/6 mice or male BALB/C nude mice purchased from Beijing Vital River Laboratory Animal Technology Company were anesthetized with sevoflurane.

For single-photon emission computed tomography (SPECT) imaging and biodistribution studies, one group of C57BL/6 mice were subcutaneously injected in the right flank with 1 × 10^6^ B16F10 cells in 50 μL of PBS. The BALB/C nude mice were subcutaneously injected in the right shoulder with A375 cells (2 × 10^6^ in 50 μL PBS).

For therapy investigation, another group of C57BL/6 mice were inoculated with 1 × 10^6^ B16F10 cells by dorsal subcutaneous injection on day 0 of the experiment.

All animals were orally administrated potassium iodide solution (0.1%) 3 days prior to the start of treatment to block the thyroid until the end of the experiment.

### Tissue biodistribution ex vivo

Six days after tumor inoculation with a volume of ~ 60 mm^3^, the C57BL/6 mice (*n* = 5 in each group) were injected with ^131^I-5-IPN (approximate 3.7 MBq, 100 μL) via a tail vein, and sacrificed at 1, 6, 24, 48 and 72 h. The organs of interest were harvested and weighed. Activity was quantified using a *γ*-counter.

### SPECT imaging

SPECT imaging was conducted to observe the whole-body distribution of ^131^I-5-IPN in C57/BL6 mice bearing B16F10 melanoma and BALB/c nude mice bearing A375 melanoma using a human SPECT/computed tomography (CT) device (Symbia T6®, Siemens, Erlangen, Germany), with two high-energy collimators. The images were acquired by two parallel detectors rotating around the mouse for 32 projections, projection/5.6°. The acquisition was 20 s per projection with a matrix of 256 × 256 pixels focusing on the right shoulder region. A whole-body CT scan (100 mA, 130 kV, 1 mm section thickness) was acquired for subsequent SPECT/CT fusion (CapGM, Siemens syngo MI Workplace, Germany). All the models were injected with ^131^I-5-IPN (3.7 MBq, 100 μL) via tail vein. For blocking study, the C57/BL6 mice bearing B16F10 melanoma were pretreated with 1000-fold non-radioactive I-5-IPN (61.5 nmol, 21.4 μg) 30 min before administrating ^131^I-5-IPN.

### Targeted radionuclide therapy study of C57BL/6 mice bearing B16F10 melanoma

This study comprised two series of experiments. The first series was to determine whether ^131^I-5-IPN inhibits tumor growth. When the B16F10 tumors reached a volume of ~ 60 mm^3^ (day 6), the mice were randomly allocated to receive one of five treatments (10 mice per treatment group) via intravenous injection as follows (*n* = 10): group A received 0.1 mL saline; group B received an equimolar dose of unlabeled precursor; group C received 18.5 MBq [^131^I]NaI; group D received 18.5 MBq ^131^I-5-IPN; group E received two doses of 18.5 MBq ^131^I-5-IPN, one on day 6 and one on day 9. To observe tumor growth, the length (L) and width (W) of the tumors were measured with electronic calipers every other day during the experiment. Tumor volume (TV) was calculated with the formula: TV (mm^3^) = (L × W^2^)/2. To evaluate the potential toxicity of ^131^I-5-IPN, body weight was recorded simultaneously. Mice were followed until end points were evidenced: tumor volumes > 1500 mm^3^; mortality; ulcerating tumor tissue; > 15% weight loss.

Subsequently, we performed histopathologic, flow cytometry, western blot, and enzyme-linked immunosorbent assay (ELISA) studies on five mice bearing B16F10 from each group. At 1 and 12 days post-injection (pi) of saline or the two doses ^131^I-5-IPN in Group E, 5 mice per group were sacrificed. The tumor tissues were harvested and divided into three parts and the specimens fixed in 10% formalin for histologic studies and immediately preserved in DMEM for flow cytometry DNA analysis or stored at − 80 °C for molecular studies. Additionally, to further investigate whether TRNT with ^131^I-5-IPN has toxic effects on normal organs, before the mice were sacrificed, blood samples were first collected by removing the eyes under anesthesia for white blood cell (WBC), red blood cell (RBC) and platelet counts, liver function (alanine aminotransferase, ALT) and renal function test (blood urea nitrogen, BUN). The livers and kidneys were collected and fixed in 10% formalin for histological analyses.

### Histopathology examination and immunofluorescence

Histopathology examination was performed on B16F10 tumors, livers, and kidney according to our previous work [23]. Stained sections of livers and kidneys were evaluated for necrosis, apoptosis, and inflammatory changes under light microscopy.

Immunofluorescence staining for the cell proliferation antibody Ki67 and the cell junction protein cluster of differentiation 31(CD31) was performed on B16F10 tumor sections to evaluate tumor cell proliferation and vascularization. Briefly, tumor sections were blocked in 5% bovine serum albumin for 20 min and incubated in rabbit anti-human Ki67 (1:200, SanYing) and rat anti-mouse CD31(1:150, Abcam) overnight at 4 °C. After washing with PBS, these sections were incubated with Cy3-conjugated goat anti-rabbit and FITC-conjugated goat anti-rat secondary antibodies (1:50; Aspen), respectively, for 50 min at 37 °C. Last, the slides were imaged with fluorescence microscopy (MicroPublisher Microscope®, QImaging, Canada).

### Flow cytometry DNA analysis

Flow cytometry DNA analysis was performed on cell suspensions prepared mechanically and enzymatically from B16F10 tumor samples. Briefly, tumor tissue was made into a constitution homogenate in a tube, filtered with a 100 μm nylon filter, and centrifuged for 5 min at 300 g. The supernatant was discarded and the cells were fixed with cold 75% ethanol overnight at 4 °C. After removing the ethanol, the cells were resuspended in 100 μL RNase A, and 400 μL propidium iodide was added. After 30 min at 4 °C in the dark, the cell cycle was analyzed with a flow cytometer (FACSCalibur®, BD, USA).

### Western blot analysis and enzyme-linked immunosorbent assay

Proliferating cell nuclear antigen (PCNA) protein expression in tumor was assessed by western blot. A sample of 50 mg of minced tumor tissue was homogenized and resuspended in lysis buffer for protein extraction. Total protein concentrations were determined using a commercial bicinchoninic acid kit (ASPEN, China). Equal amount of protein (40 μg) was separated by SDS-PAGE and transferred to nitrocellulose membranes (Merck Millipore, Germany). Anti-PCNA antibody (1:1000, Abcam, USA) was used for immunoreaction. Goat anti-mouse antibody (1:10000) was used as a secondary antibody. The antigen was detected with a chemiluminescence detection kit (Super Signal®, ASPEN, China). Vascular endothelial growth factor (VEGF) concentration was determined using ELISA according to the manufacturer’s instruction (Mouse VEGF ELISA kit®, Neobioscience, China).

### Radiation absorbed dose of ^131^I-5-IPN

The radiation absorbed doses of ^131^I-5-IPN for humans were estimated from the biodistribution data of B16F10 tumor models (Table 1) using OLINDA/EXM program (Version 1.1).

**Table 1:** Biodistribution of ^131^I-5-IPN in B16F10 melanoma bearing C57BL/6 mice and A375 melanoma bearing BALB/C-nu/nu mice at different time pi. (% ID/g, mean ± SD)

	B16F10 melanoma (*n* = 5)					Blocked B16F10 melanoma (*n* = 5)	A375 melanoma (*n* = 5)
	1 h	6 h	24 h	48 h	72 h	1 h	1 h
Organ							
Blood	4.05 ± 1.06	1.44 ± 0.35	0.05 ± 0.01	0.04 ± 0.02	0.03 ± 0.03	0.07 ± 0.02	0.09 ± 0.02
Brain	0.94 ± 0.21	0.11 ± 0.01	0.02 ± 0.02	0.01 ± 0.01	0.01 ± 0.00	0.06 ± 0.01	0.04 ± 0.02
Heart	2.20 ± 0.38	0.47 ± 0.11	0.24 ± 0.16	0.06 ± 0.05	0.04 ± 0.04	0.11 ± 0.03	0.15 ± 0.05
Lungs	5.82 ± 4.34	1.35 ± 0.18	0.36 ± 0.17	0.27 ± 0.09	0.08 ± 0.10	0.16 ± 0.03	0.19 ± 0.04
Liver	19.81 ± 0.38	4.12 ± 0.30	1.53 ± 0.25	0.82 ± 0.28	0.46 ± 0.48	1.35 ± 0.20	1.52 ± 0.25
Spleen	4.06 ± 1.50	0.87 ± 0.09	0.39 ± 0.26	0.33 ± 0.30	0.05 ± 0.06	0.34 ± 0.15	0.11 ± 0.02
Kidney	5.87 ± 1.74	1.14 ± 0.15	0.18 ± 0.07	0.11 ± 0.04	0.05 ± 0.03	0.61 ± 0.53	0.17 ± 0.01
Stomach	5.22 ± 1.55	2.24 ± 1.43	0.08 ± 0.02	0.11 ± 0.10	0.05 ± 0.03	0.17 ± 0.09	0.51 ± 0.28
Large intestine	3.43 ± 0.52	0.72 ± 0.36	0.11 ± 0.06	0.06 ± 0.06	0.05 ± 0.06	0.29 ± 0.07	0.17 ± 0.04
Small intestine	3.56 ± 0.40	0.79 ± 0.47	0.05 ± 0.01	0.05 ± 0.03	0.02 ± 0.01	0.38 ± 0.18	0.18 ± 0.03
Muscle	1.18 ± 0.33	0.28 ± 0.03	0.02 ± 0.02	0.01 ± 0.00	0.02 ± 0.01	0.07 ± 0.02	0.17 ± 0.04
Bone	3.24 ± 1.50	0.65 ± 0.08	0.12 ± 0.03	0.56 ± 0.89	0.04 ± 0.07	0.16 ± 0.01	0.17 ± 0.05
Tumor	16.37 ± 3.32	14.33 ± 3.30	7.41 ± 2.21	4.26 ± 1.36	3.31 ± 1.45	2.67 ± 1.16	0.15 ± 0.12
Thyroid	6.51 ± 2.13	1.36 ± 0.17	0.11 ± 0.04	0.08 ± 0.05	0.04 ± 0.06	0.29 ± 0.08	0.25 ± 0.02
Eyes	22.45 ± 4.74	15.02 ± 2.12	11.27 ± 0.90	9.77 ± 1.02	7.17 ± 0.96	4.66 ± 1.10	0.30 ± 0.02
Uptake ratio							
Tumor-to-blood	4.16 ± 0.74	10.04 ± 0.75	151.3 ± 64.35	132.9 ± 124.9	188.1 ± 110.9	50.56 ± 40.23	1.49 ± 1.02
Tumor-to-liver	0.85 ± 0.18	3.47 ± 0.70	4.91 ± 1.65	5.45 ± 2.16	10.49 ± 5.01	1.96 ± 0.73	0.10 ± 0.07
Tumor-to-muscle	14.53 ± 3.33	51.72 ± 13.58	248.4 ± 143.7	121.5 ± 66.76	123.4 ± 37.89	40.08 ± 16.20	0.82 ± 0.29

### Statistical analysis

We used commercial software (GraphPad Prism® version 5.01, GraphPad Software, Inc., USA), performing a two-tailed Student *t*-test for comparing means of two independent groups and one-way analysis of variance (ANOVA) and post hoc (Tukey’s) tests when multiple comparisons were needed. In the treatment experiments, survival was assessed by the Kaplan-Meier method. A value of *P* < 0.05 was considered statistically significant.

## Results

### Chemical and radiochemical

**Fig. 1:**
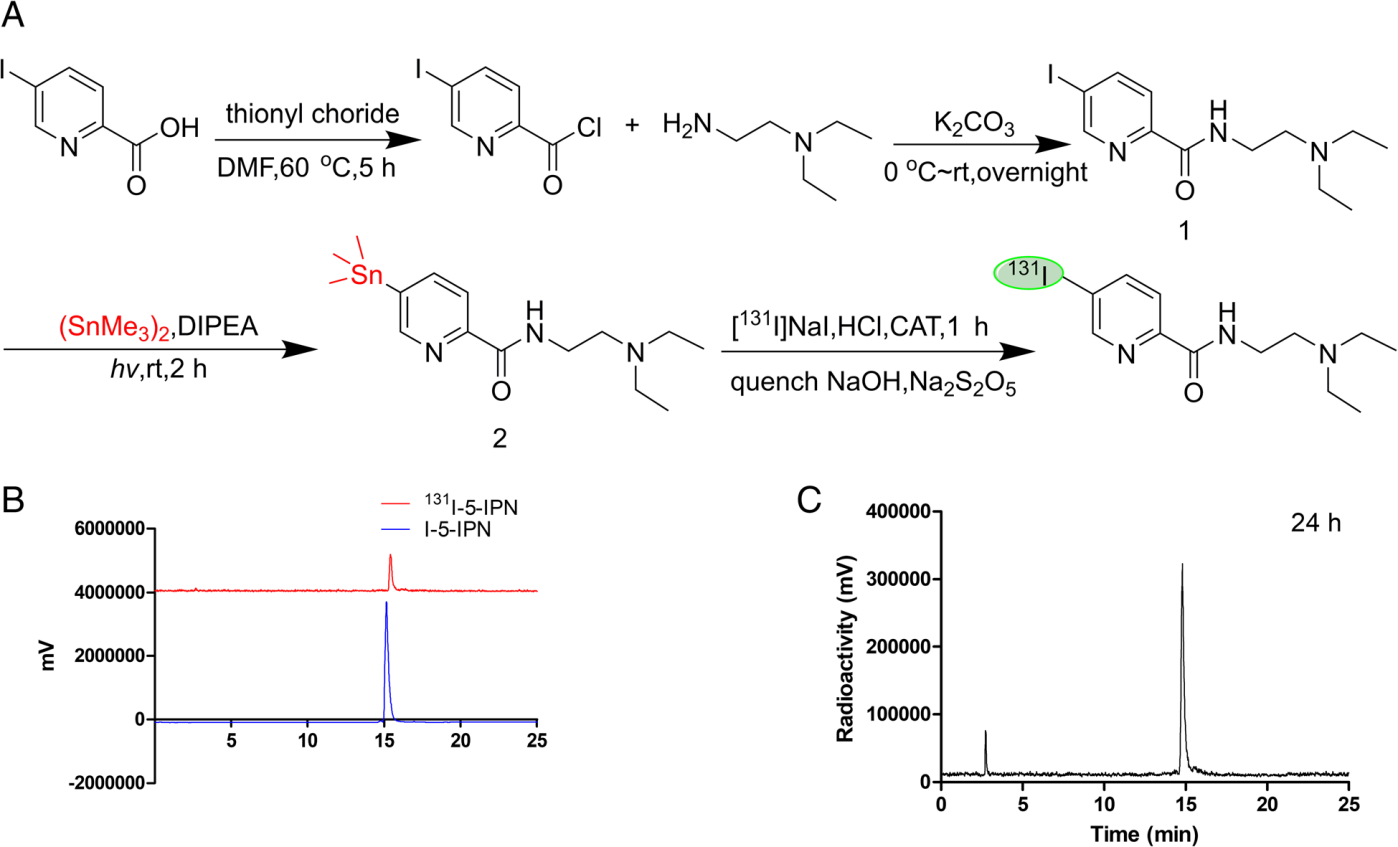
**a** Synthesis of ^131^I-5-IPN and its trimethylstannyl precursor. **b** Analytic high-pressure liquid chromatography of ^131^I-5-IPN (red, radio peak, retention time 15.38 min) and I-5-IPN (blue, UV-peak, 254 nm). **c** ^131^I-5-IPN stability in human serum post incubation at 37 °C for 24 h

As shown in Fig. 1a, the synthesis of the trimethylstannyl precursor for radiolabeling can be achieved via an efficient photo-induced transition-metal-free stannylation reaction. ^131^I-5-IPN was readily labeled with ^131^I using a radioiododestannylation reaction, identified by co-eluting with the nonradioactive compound 1 (Fig. 1b). The tracer was synthesized with a good radiochemical yield (50–60%), high radiochemical purity (> 98%), and specific activity (5.45–6.55 GBq/μmol).

As shown in Fig. 1c, ^131^I-5-IPN displayed good stability in human serum with no obvious deiodination for up to 24 h. The percentage of intact ^131^I-5-IPN was > 96% after incubation for 24 h at 37 °C.

Log *P* is usually used to reflect the lipophilic or hydrophilic properties of a compound. A Log *P* value of 0.03 ± 0.03 revealed that the distribution of ^131^I-5-IPN in two phases is slightly lipophilic.

### In vitro cell study

**Fig. 2:**
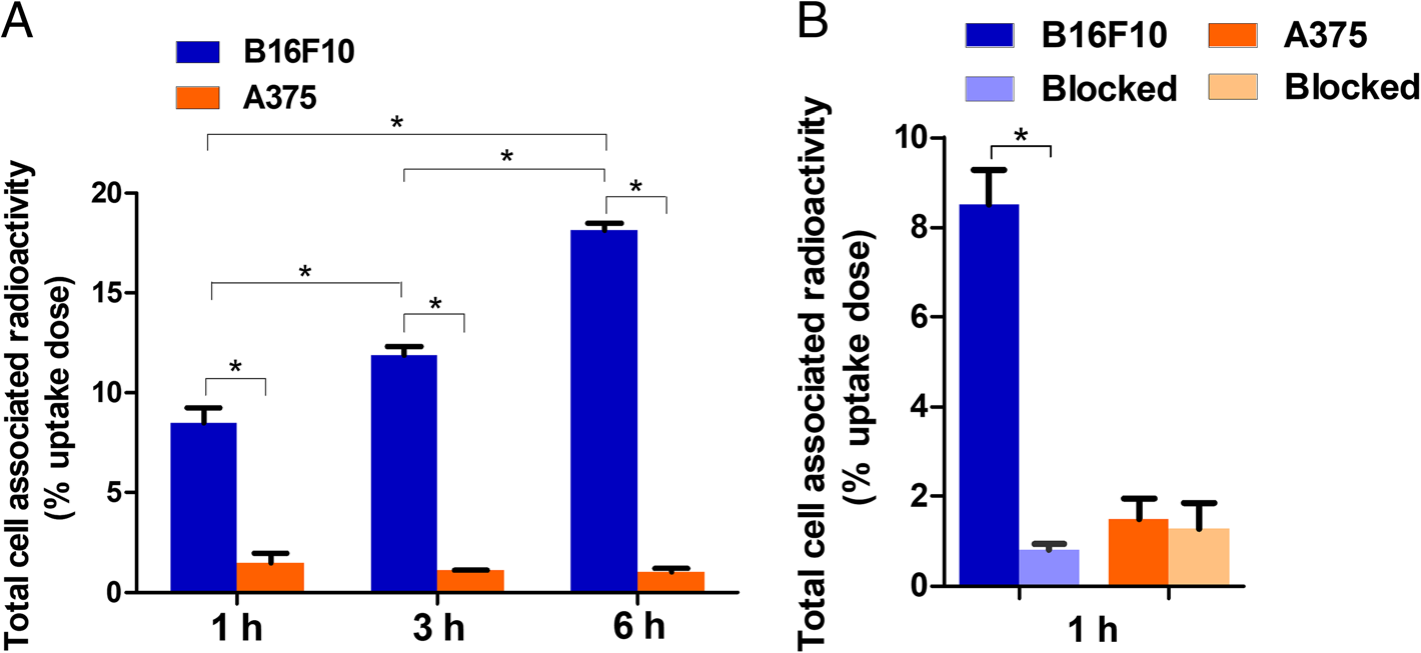
**a** In vitro cell uptake of ^131^I-5-IPN by pigmented B16F10 cells and amelanotic A375 cells at 1, 3, and 6 h. The uptake of ^131^I-5-IPN showed a time-dependent increase trend in B16F10 cells (*P < 0.001). The uptakes of ^131^I-5-IPN in B16F10 cells were significantly higher than those in A375 cells at all time points (*P < 0.001). **b** Blocking study of ^131^I-5-IPN pretreated with nonradiolabeled I-5-IPN in B16F10 cells and A375 cells. The uptake of ^131^I-5-IPN showed statistical decreased in B16F10 cells after pretreated with 1000-fold non-radioactive I-5-IPN (*P < 0.001)

As Fig. 2a demonstrates, ^131^I-5-IPN showed significant uptake by pigmented B16F10 cells at 1, 3, and 6 h (8.50 ± 0.77%, 11.88 ± 0.46% and 18.16 ± 0.35%, respectively) and showed a time-dependent increase trend. In contrast, the uptake by A375 cells was obviously lower (1.48 ± 0.47%, 1.12 ± 0.03% and 1.04 ± 0.17%, respectively). The uptakes of ^131^I-5-IPN in B16F10 cells were significantly higher than those in A375 cells (*P < 0.001). Figure 2b shows the uptake reduction B16F10 cells by pretreatment with excess nonradioactive I-5-IPN, but which did not markedly change uptake by A375 cells.

### In vivo pharmacokinetic and tumor targeting properties of ^131^I-5-IPN

**Fig. 3:**
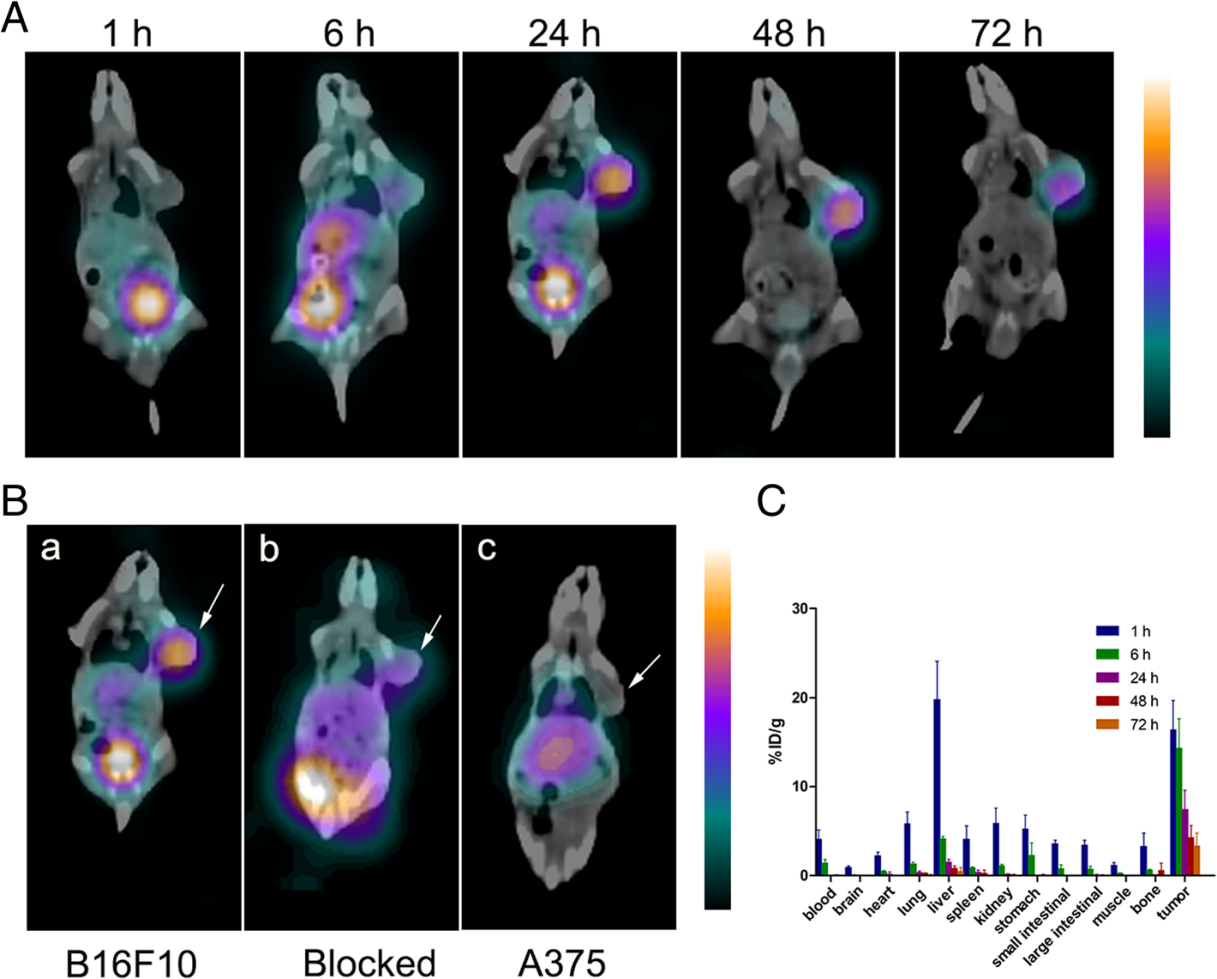
**A** SPECT imaging of ^131^I-5-IPN in B16F10 melanoma bearing C57BL/6 mice at 1, 6, 24, 48 and 72 h. **B** Representative SPECT images of melanoma models at 24 h pi. (a) B16F10 tumor-bearing mouse, (b) B16F10 tumor-bearing mouse pretreated with nonradiolabeled I-5-IPN and (c) A375 tumor xenograft bearing BALB/c nude mouse. Arrows indicate tumor. **C** Biodistribution analyses of ^131^I-5-IPN in B16F10 melanoma bearing C57BL/6 mice, at 1 h, 6 h, 24 h, 48 h and 72 h

As shown in Fig. 3A, the B16F10 tumors were clear and identifiable in SPECT imaging at 6, 24, 48 and 72 h. At 48 h, tumors could be clearly visualized with low background, and the uptake in liver and normal tissues had become undetectable. These results were consistent with the biodistribution data (Fig. 3c, Table 1). At 1 h post ^131^I-5-IPN injection, radioactivity rapidly accumulated in the liver (19.81 ± 0.38% ID/g) but decreased to 4.12 ± 0.30% ID/g at 6 h pi. The tumor could not be clearly visualized in SPECT imaging until 24 h; the high accumulation in liver and bladder most likely accounts for this finding. With ^131^I-5-IPN gradually clearing from nontarget organs, the visibility of the tumor becomes clearer, showing a prolonged retention (i.e., 7.41 ± 2.21% ID/g at 24 h; 4.26 ± 1.36% ID/g at 48 h; 3.31 ± 1.45% ID/g at 72 h). In contrast, the uptake in amelanotic A375 tumor was lower (Fig. 3B-c; Table 1), and uptake in B16F10 tumor pretreated with excess nonradiolabeled I-5-IPN also showed significantly lower intensity 24 h pi of ^131^I-5-IPN (Fig. 3B-b; Table 1).

### ^131^I-5-IPN inhibits the growth of B16F10 tumors and prolongs survival

**Fig. 4:**
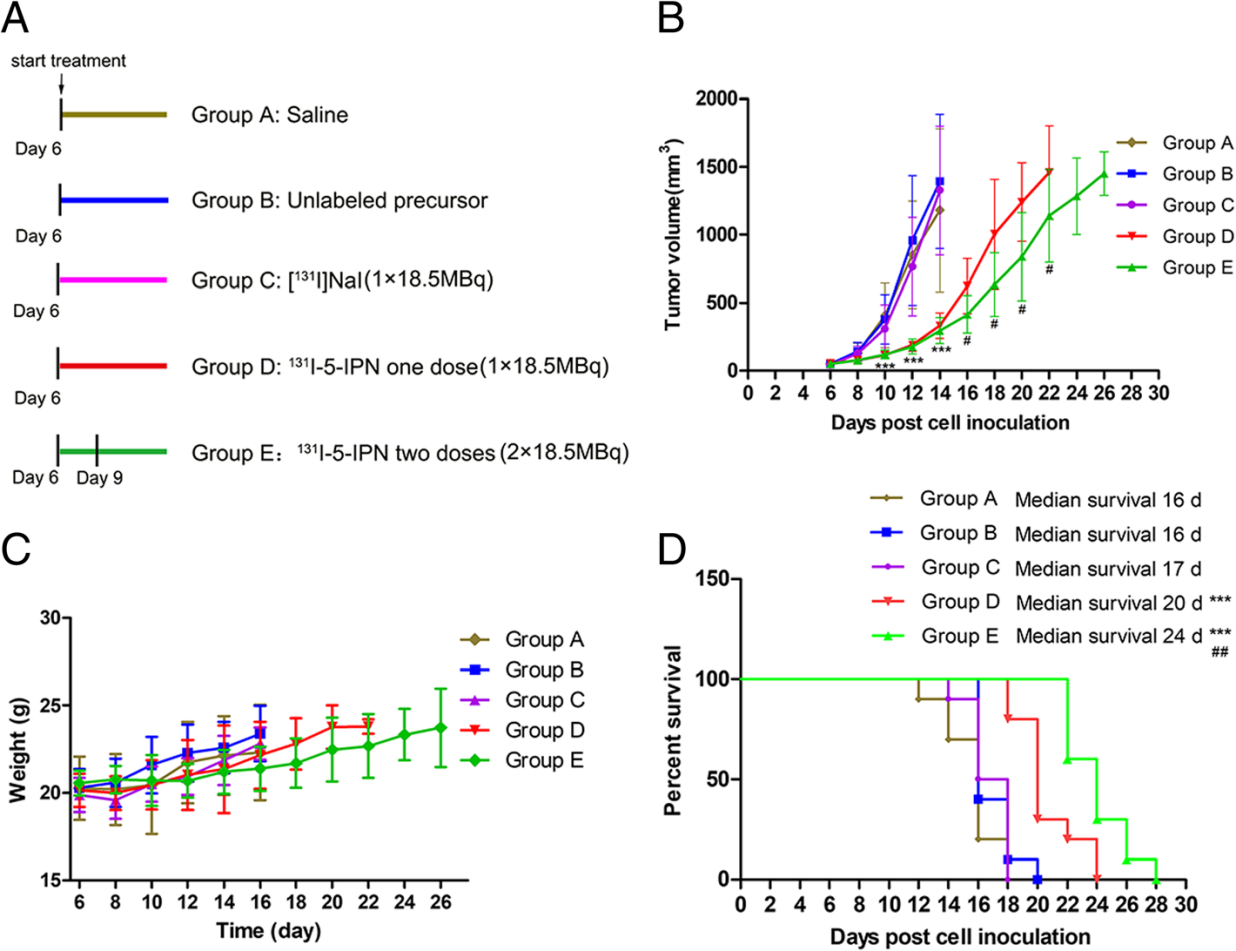
**a** Therapy experimental protocol. **b** Changes in mean tumor volume. TRNT with ^131^I-5-IPN resulted in a significant delay in tumor growth (***P < 0.001, compared with group A; #*P* < 0.05, compared with group D). **c** Body weight changes during treatment (*P >* 0.05). **d** Kaplan-Meier survival plot in B16F10 models. (***P < 0.001, compared with group A. ##*P* < 0.01, compared with group D)

The antitumor effect of ^131^I-5-IPN is shown in Fig. 4. As shown in Fig. 4b and d, both single-dose (group D) and double-dose (group E) treatment with ^131^I-5-IPN dramatically inhibited tumor growth 10 days after therapy (*P* < 0.001), and double-dose was more effective than the single-dose. Median survival in groups D (20 d) and E (24 d) was significantly extended when compared to saline (16 d), unlabeled precursor (16 d) and [^131^I]NaI (17 d) (*P* < 0.001). Figure 4c shows that the weight of the mice in each group increased steadily, but with no statistical significance among the groups (*P* > 0.05).

### Biologic effects of TRNT in B16F10 tumor

**Fig. 5:**
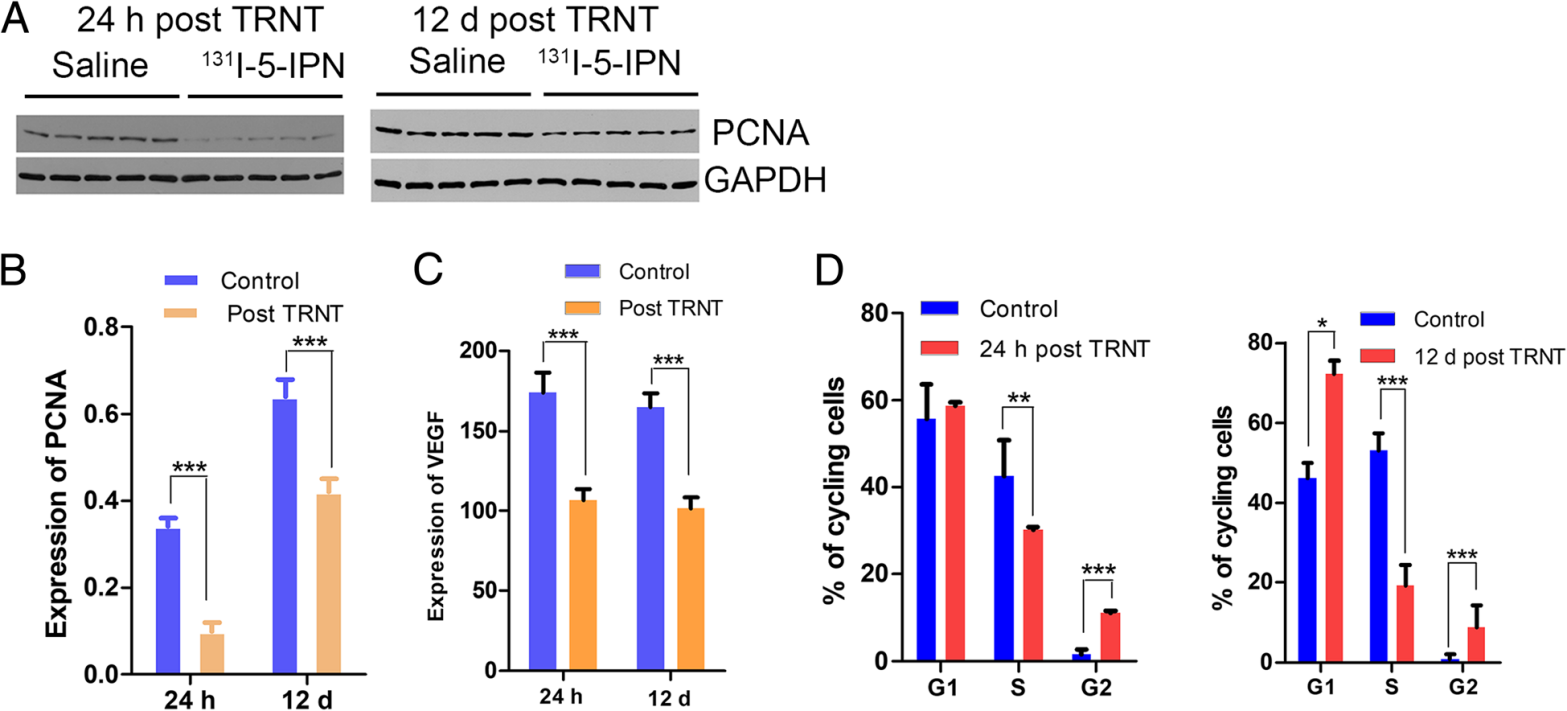
Biologic characterization of TRNT with ^131^I-5-IPN in B16F10 model. **a** 24 h and (**b**) 12 d post-TRNT western blot and quantitative analysis of PCNA in tumors. **c** ELISA analysis of VEGF at 24 h and 12 d post TRNT. **d** Cell cycle analysis of tumor cells at 24 h and 12 d post-TRNT (*P < 0.05, **P < 0.001, ***P < 0.0001)

**Fig. 6:**
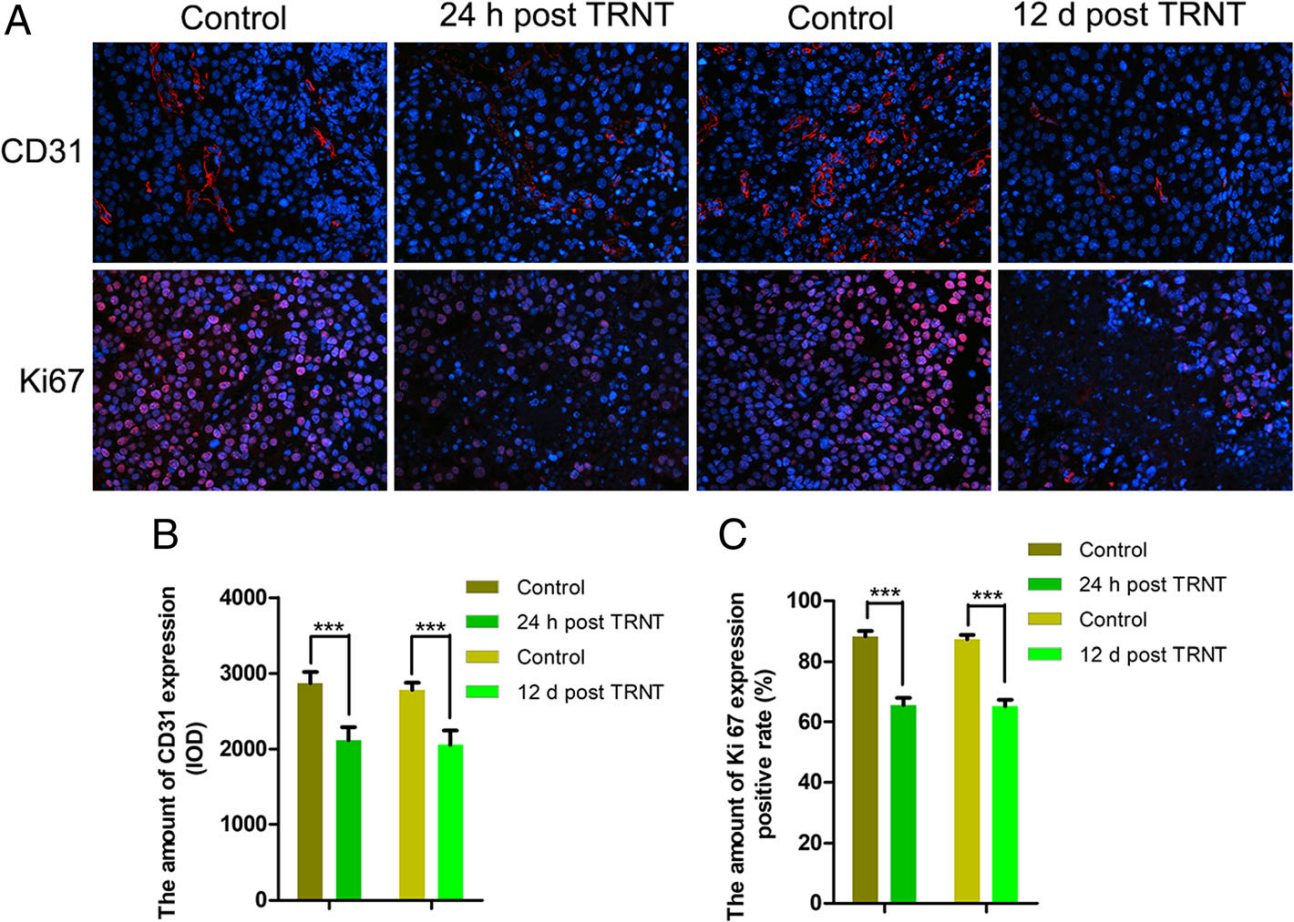
Biologic characterization of TRNT with ^131^I-5-IPN in B16F10 model. **a** Immunofluorescence staining for CD31 (vascularity) and Ki67 (proliferation) in tumors 24 h and 12 d after TRNT (× 200). **b** and **c** The amount of CD31 expression and Ki67 expression quantified from immunofluorescence staining of treated and control tumors (***P < 0.0001)

To evaluate tumor proliferation, PCNA expression, Ki67, expression and cell cycle were analyzed by western blot, immunofluorescence staining, and flow cytometry. CD31 and VEGF expression levels were also measured using immunofluorescence staining or ELISA to evaluate the tumor vasculature. As shown in Fig. 5a and b, PCNA protein levels were significantly decreased at both 24 h and 12 days post-TRNT. A relatively high portion of cells in the saline control group stained positively for Ki67 (Fig. 6a and c), whereas significantly reduced cell proliferation was observed in treated group as early as 24 h post-TRNT. Similarly, the cell cycle was blocked in the G2 phase in the treated animals, but 42 and 51% cell accumulation was noted in S phase in the saline group at 24 h and 12 days (Fig. 5d), indicating rapid proliferation of tumor cells.

Compared with the saline group, the treated animals had a significant decrease in VEGF protein levels at 24 h and 12 days (Fig. 5c). CD31 immunofluorescence analysis also showed significantly decreased blood vessel density in both treatment groups (Fig. 6a and b).

### Toxicity of TRNT in normal organs

**Fig. 7:**
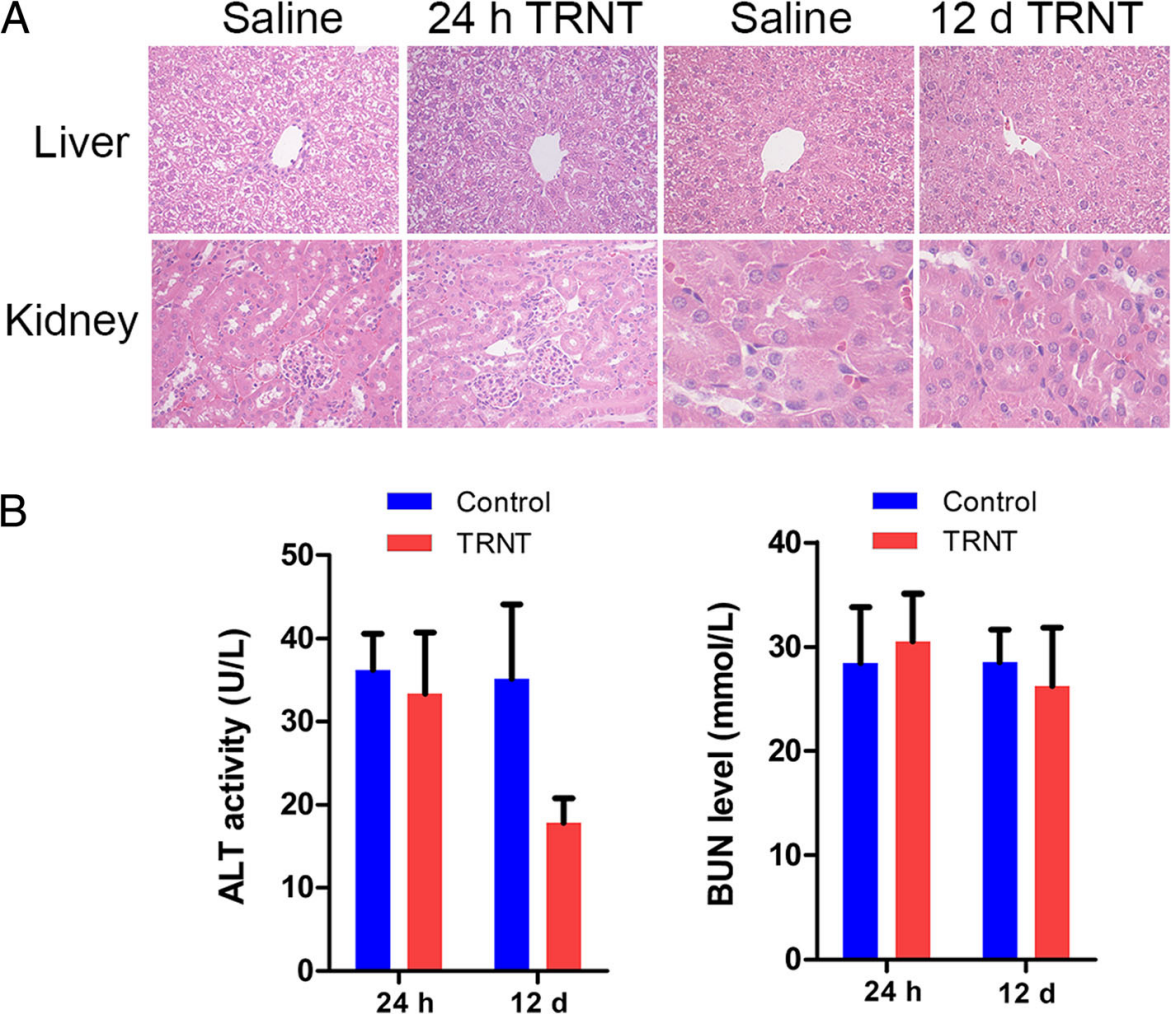
Toxicity assessment of vital organs after TRNT with ^131^I-5-IPN. **a** H&E staining of liver and kidneys at 24 h and 12 d post-treatment in control and treatment group (× 200). **b** ALT activity and BUN level of mice injected with ^131^I-5-IPN (2 × 18.5 Bq) in Group E and Group A (saline control) at 24 h and 12 d pi

In view of high uptake by the liver and kidneys (main excretory organ), toxicity associated with ^131^I-5-IPN was monitored by pathology (Fig. 7a). Pathology tests showed no evidence of toxicity in the kidneys and livers 24 h and 12 d pi of two doses of ^131^I-5-IPN (Group E). Likewise, no difference was found in serum BUN and ALT 24 h and 12 d post-TRNT (Fig. 7b). The hematological toxicity of ^131^I-5-IPN TRNT was assessed by measuring white blood cell, red blood cell, and platelet counts at 24 h and 12 days pi. There was no significant difference in WBC, RBC and platelet counts (Table 2).

**Table 2:** WBC, RBC, and platelet counts of mice injected with ^131^I-5-IPN (2 × 18.5 Bq) (TRNT) and saline (control) at 24 h and 12 d pi

Time (h/d)	RBC count (× 10^12^/L)		WBC count (× 10^9^/L)		Platelet count (× 10^9^/L)	
	Control	TRNT	Control	TRNT	Control	TRNT
24 h	5.10 ± 0.32	5.32 ± 0.52	9.05 ± 0.69	9.57 ± 0.44	511.8 ± 23.75	507.2 ± 43.31
12 d	5.31 ± 0.62	5.11 ± 0.62	9.23 ± 0.80	8.64 ± 0.78	508.0 ± 30.58	527.1 ± 47.38

### Radiation absorbed dose of ^131^I-5-IPN

The simulated dosimetry to humans is shown in Table 3. The radiation absorbed dose was highest in liver (around 0.25 mSv/MBq). The absorbed doses in kidneys (0.17 mSv/MBq), lungs (0.11 mSv/MBq) and brain (0.01 mSv/MBq) were low, and that in red marrow (0.003 mSv/MBq) was insignificant.

**Table 3:** Simulated radiation absorbed dose to humans extrapolated from ^131^I-5-IPN in B16F10 melanoma-bearing C57BL/6 mice

Organs	Radiation absorbed dose (mSv/MBq)
Brain	1.27E-02
Heart	1.08E-01
Lungs	1.29E-01
Liver	2.50E-01
Spleen	1.25E-01
Kidney	1.73E-02
Stomach	1.45E-02
Red marrow	3.43E-03
Osteogenic cells	9.11E-03
Effective Dose	9.08E-02

Extrapolated radiation absorbed dose for a 70 kg male adult

## Discussion

Since more than 90% of primary melanomas [25] and 50% of their metastases [26, 27] contain melanin, melanin-targeting TRNT is a promising new therapy option for advanced metastatic melanoma. In our previous study, the picolinamide probe ^18^F-5-FPN displayed high specificity and affinity for intracellular melanin as well as rapid whole-body elimination [22, 28]. In this study, we further prepared ^131^I-5-IPN, which shows high tumor uptake, lasting tumor retention and rapid whole-body clearance in melanin-positive B16F10 models. TRNT with ^131^I-5-IPN presents strong anti-tumor efficacy without evident toxicity, suggesting that ^131^I-5-IPN has the potential for TRNT for melanoma.

In the in vitro cell studies, ^131^I-5-IPN exhibited a high uptake ratio by melanin-positive B16F10 cells and low, nonspecific uptake by amelanotic A375 cells, indicating high specificity. SPECT imaging and biodistribution of ^131^I-5-IPN showed lasting high tumor uptake by pigmented B16F10 tumors from 1 h to 72 h pi. In theory, a lipophilic compound will be excreted mainly by the hepatobiliary system, while hydrophilic compounds will be excreted mostly through the urinary system. A log *P* of 0.03 ± 0.03 predicts ^131^I-5-IPN is a bit more lipophilic than ^18^F-5-FPN (Log *P* = − 0.17 ± 0.01) [23], meaning ^131^I-5-IPN would distribute more in liver than ^18^F-5-FPN. Indeed, ^131^I-5-IPN accumulated in liver with 19.81 ± 0.38% ID/g in the inception phase. Fortunately, the high uptake of ^131^I-5-IPN in liver was temporary and decreased to 4.12 ± 0.30% ID/g at 6 h pi. Moreover, uptake by the kidneys was very low at 24 h pi, which is superior to other melanin-targeting molecules such as mAbs and peptides [9–13]. Overall, ^131^I-5-IPN exhibited good in vivo behavior for SPECT imaging and TRNT, with high and prolonged tumor uptake and relatively rapid clearance from non-target organs (Table 1).

We further evaluated the potential therapeutic ability of ^131^I-5-IPN in pigmented B16F10 melanoma mouse models. The study demonstrated that both a single dose and two doses of ^131^I-5-IPN significantly slowed tumor growth and prolonged median survival time (20 d and 24 d, respectively), whereas both unlabeled precursor alone and [^131^I]NaI alone had no effect on tumor growth and median survival (16 d and 17 d, respectively). These results revealed that a targeted carrier molecule possessing high affinity and specificity is crucial for TRNT. Theoretically, multiple doses have more advantages than a single dose. First, multiple doses can avoid an overdose of side effects. Second, multiple doses can maintain higher drug concentration in the target tissue during treatment. Third, the dosing interval allows enough time to remove from nontarget organs (for example, liver) in case of unnecessary radiation damage. In the current experiment, we selected 3 d as the dosing interval on the basis of biodistribution data and SPECT imaging. Two doses (2 × 18.5 MBq) of ^131^I-5-IPN were more effective and exhibited longer growth delay than a single dose (Fig. 5b and d), and no systemic toxic effect was observed from weight changes.

The molecular biology study further confirmed the strong TRNT efficacy of ^131^I-5-IPN. We observed decreased expression of proliferation marker PCNA and Ki67 at 24 h and 12 d pi; meanwhile, we also found radiation-induced DNA damage through cell cycle analyses of treated tumor. These changes suggested tumors lose aggressiveness after TRNT. CD31 immunofluorescence analysis also confirmed significantly decreased blood vessel density in both groups after TRNT. Consistent with this, VEGF expression in tumor was strongly downregulated after 12 days pi.

It is important to evaluate the radiotoxicity of ^131^I-5-IPN. We did not observe significant changes in WBC, RBC or platelet counts at 24 h and 12 days pi. It is known that ALT level usually rises when the liver is being damaged, but no elevated ALT level was observed in the treated mice, and no injury was found in hepatic pathology at 24 h and 12 d post-treatment. Additionally, the kidneys as the main excretory organs were also unaffected. Importantly, this study was performed on C57BL/6 mice with high ocular melanin content, as seen by high uptake in pigmented eyes with ^18^F-5-FPN [22]. This is a concern for therapeutic applications. The melanin content level in ocular tissues differs among species, even within different structures in the same organ [29]. In the clinical trial with [^123^I]BZA2, there was no significant radioactivity in the eyes of patients [30]. Instead, high uveal radioactivity was detected with BZA2 in C57BL/6 mice [31]. Moreover, another agent targeting melanin, ^131^I-MIP-1145, showed more than 30% ID/g radioactivity in the eyes of C57BL/6 mice, but the highest radiation-absorbed dose was only 6.8 Gy when a 3.7 GBq therapeutic dose was injected into *Cynomolgus* monkey [20]. External beam data showed no significant retinopathy in standard fractionated treatment when maximum dosage was < 50 Gy [32]. Therefore, these clinical experimental results and differences between murine and human in eye structure and melanin content are enough to prove that ^131^I-5-IPN uptake in the eyes does not appear to be a major stumbling block for further clinical application.

In order to obtain more valuable toxicological assessment for TRNT of melanoma, the radiation dosimetry of ^131^I-5-IPN was calculated. The estimated radiation absorption doses to vital nontarget organs from ^131^I-5-IPN, for example, liver (0.25 mSv/MBq), kidneys (0.02 mSv/MBq) and lungs (0.13 mSv/MBq) were far lower than those reported in a clinical trial with ^131^I-BA52 (1.30, 3.15 and 0.30 Gy/GBq, respectively) [21] for TRNT of patients with metastatic melanoma. These satisfied absorbed doses in nontarget and target organs suggested that ^131^I-5-IPN is a potential targeted therapeutic agent for patients with melanoma.

Some aspects of this work should be noted as limitations. First, this study was performed in murine pigmented B16F10 models, and further study should be conducted in mice bearing xenografted human pigmented melanoma. Second, B16Bl6 cells are more aggressive and can generate spontaneous lung metastases from primary subcutaneous tumor, providing a metastatic model for further therapy; they should be evaluated in mice. Third, the relative high uptake in the liver at first several hours was noticed. More work should be done for the modification of the small molecular and it’s radiolabeling method to optimize the pharmacokinetics in vivo. Forth, more accurate human dosimetry must be established through SPECT imaging studies of human subjects. More attention should be paid to the radiation absorbed dose of the eye. Last but not least, it is clear that a monotherapy regimen is seldom successful due to the highly aggressive biological nature of melanoma. A combination of CTLA-4 inhibitors and anti-PD-1 agents has been shown to prolong overall survival in patients with advanced melanoma [33]. Again, radiotherapy has tangible efficacy in priming the immune response such as reducing the overexpression of ligands inhibiting the host’s antitumor immunity and secretion of immunosuppressive cytokines [34, 35]. It is speculated that ^131^I-5-IPN combined with CTLA-4 or PD-1 inhibitors may be a promising therapeutic direction for patients with metastatic melanoma.

## Conclusion

In this study, ^131^I-5-IPN was synthesized with a substantial yield. The TRNT study demonstrated that treatment with ^131^I-5-IPN significantly slows down tumor growth and prolonged median survival of B16F10 melanoma models. No obvious toxicity in normal organs was observed during treatment. ^131^I-5-IPN appears to be a potential TRNT agent for melanoma; also it could be used as a theranostic agent.

## Abbreviations

CT: Computed tomography
DCM: Dichloromethane
DMEM: Dulbecco’s modified Eagle’s medium
ELISA: Enzyme-linked immunosorbent assay
HPLC: High-pressure liquid chromatography
mAb: Monoclonal antibody
MeCN: Acetonitrile
MeOH: Methanol
PBS: Phosphate-buffered saline
PCNA: Proliferating cell nuclear antigen
PET: Positron-emission tomography
SPECT: Single-photon emission computed tomography
TRNT: Targeted radionuclide therapy
TV: Tumor volume
VEGF: Vascular endothelial growth factor

## References

[1] Karimkhani C, Green AC, Nijsten T (2017). The global burden of melanoma: results from the global burden of disease study 2015. Br J Dermatol.

[2] Karimkhani C, Reddy BY, Dellavalle RP (2017). Novel therapies for unresectable and metastatic melanoma. BMJ.

[3] Huncharek M, Caubet JF, Mcgarry R (2001). Single-agent DTIC versus combination chemotherapy with or without immunotherapy in metastatic melanoma: a meta-analysis of 3273 patients from 20 randomized trials. Melanoma Res.

[4] Espenel S, Vallard A, Rancoule C (2017). Melanoma: last call for radiotherapy. Crit Rev Oncol Hematol.

[5] Norain A, Dadachova E (2016). Targeted radionuclide therapy of melanoma. Semin Nucl Med.

[6] Strosberg J, El-Haddad G, Wolin E (2017). Phase 3 trial of 177Lu-Dotatate for midgut neuroendocrine tumors. N Engl J Med.

[7] Witzig TE (2003). Efficacy and safety of 90Y ibritumomab tiuxetan (Zevalin) radioimmunotherapy for non-Hodgkin's lymphoma. Semin Oncol.

[8] Kaminski MS, Tuck M, Estes J (2005). 131I-tositumomab therapy as initial treatment for follicular lymphoma. N Engl J Med.

[9] Scott AM, Lee FT, Hopkins W (2001). Specific targeting, biodistribution, and lack of immunogenicity of chimeric anti-GD3 monoclonal antibody KM871 in patients with metastatic melanoma: results of a phase I trial. J Clin Oncol.

[10] Dadachova E, Revskaya E, Sesay MA (2008). Pre-clinical evaluation and efficacy studies of a melanin-binding IgM antibody labeled with 188Re against experimental human metastatic melanoma in nude mice. Cancer Biol Ther.

[11] Miao Y, Owen NK, Fisher DR (2005). Therapeutic efficacy of a 188Re-labeled alpha-melanocyte-stimulating hormone peptide analog in murine and human melanoma-bearing mouse models. J Nucl Med.

[12] Howell RC, Revskaya E, Pazo V (2007). Phage display library derived peptides that bind to human tumor melanin as potential vehicles for targeted radionuclide therapy of metastatic melanoma. Bioconjug Chem.

[13] Beaino W, Nedrow JR, Anderson CJ (2015). Evaluation of (68)Ga- and (177)Lu-DOTA-PEG4-LLP2A for VLA-4-targeted PET imaging and treatment of metastatic melanoma. Mol Pharm.

[14] Gai Y, Sun L, Lan X (2018). Synthesis and evaluation of new bifunctional chelators with Phosphonic acid arms for Gallium-68 based PET imaging in melanoma. Bioconjug Chem.

[15] Denoyer D, Greguric I, Roselt P (2010). High-contrast PET of melanoma using (18)F-MEL050, a selective probe for melanin with predominantly renal clearance. J Nucl Med.

[16] Liu H, Liu S, Miao Z (2013). Development of 18F-labeled picolinamide probes for PET imaging of malignant melanoma. J Med Chem.

[17] Rbah-Vidal L, Vidal A, Billaud EM (2017). Theranostic approach for metastatic pigmented melanoma using ICF15002, a multimodal radiotracer for both PET imaging and targeted radionuclide therapy. Neoplasia.

[18] Garg PK, Nazih R, Wu Y (2017). 4-11C-Methoxy N-(2-Diethylaminoethyl) Benzamide: a novel probe to selectively target melanoma. J Nucl Med.

[19] Bonnet-Duquennoy M, Papon J, Mishellany F (2009). Targeted radionuclide therapy of melanoma: anti-tumoural efficacy studies of a new 131I labelled potential agent. Int J Cancer.

[20] Joyal JL, Barrett JA, Marquis JC (2010). Preclinical evaluation of an 131I-labeled benzamide for targeted radiotherapy of metastatic melanoma. Cancer Re.

[21] Mier W, Kratochwil C, Hassel JC (2014). Radiopharmaceutical therapy of patients with metastasized melanoma with the melanin-binding benzamide 131I-BA52. J Nucl Med.

[22] Feng H, Xia X, Li C (2016). Imaging malignant melanoma with 18F-5-FPN. Eur J Nucl Med Mol Imaging.

[23] Xu X, Yuan L, Yin L (2017). Synthesis and preclinical evaluation of 18F-PEG3-FPN for the detection of metastatic pigmented melanoma. Mol Pharm.

[24] Chen K, He P, Zhang S (2016). Synthesis of aryl trimethylstannanes from aryl halides: an efficient photochemical method. Chem Commun (Camb).

[25] Koch SE, Lange JR (2000). Amelanotic melanoma: the great masquerader. J Am Acad Dermatol.

[26] Ikkola J, Vihinen P, Vlaykova T (2002). High expression levels of collagenase-1 and stromelysin-1 correlate with shorter disease-free survival in human metastatic melanoma. Int J Cancer.

[27] Ghanem N, Altehoefer C, Högerle S (2005). Detectability of liver metastases in malignant melanoma: prospective comparison of magnetic resonance imaging and positron emission tomography. Eur J Radiol.

[28] Wang Y, Li M, Zhang Y (2017). Detection of melanoma metastases with PET-comparison of 18F-5-FPN with 18F-FDG. Nucl Med Biol.

[29] Durairaj C, Chastain JE, Kompella UB (2012). Intraocular distribution of melanin in human, monkey, rabbit, minipig and dog eyes. Exp Eye Res.

[30] Cachin F, Miot-Noirault E, Gillet B (2014). (123)I-BZA2 as a melanin-targeted radiotracer for the identification of melanoma metastases: results and perspectives of a multicenter phase III clinical trial. J Nucl Med.

[31] Labarre P, Papon J, Moreau MF (1999). Evaluation in mice of some iodinated melanoma imaging agents using cryosectioning and multi-wire proportional counting. Eur J Nucl Med.

[32] Emami B, Lyman J, Brown A (1991). Tolerance of normal tissue to therapeutic irradiation. Int J Radiat Oncol Biol Phys.

[33] Wolchok JD, Kluger H, Callahan MK (2013). Nivolumab plus ipilimumab in advanced melanoma. N Engl J Med.

[34] Hanna GG, Coyle VM, Prise KM (2015). Immune modulation in advanced radiotherapies: targeting out-of-field effects. Cancer Lett.

[35] Tang C, Wang X, Soh H (2014). Combining radiation and immunotherapy: a new systemic therapy for solid tumors?. Cancer Immunol Res.

