# Correction to: Targeted radiotherapy of pigmented melanoma with ^131^I-5-IPN

**DOI:** 10.1186/s13046-019-1141-z

**Published:** 2019-03-25

**Authors:** Xiaodong Xu, Lujie Yuan, Yongkang Gai, Qingyao Liu, Lianglan Yin, Yaqun Jiang, Yichun Wang, Yongxue Zhang, Xiaoli Lan

**Affiliations:** 1 Department of Nuclear Medicine, Union Hospital, Tongji Medical College, Huazhong University of Science and Technology, Wuhan, China; 2 Hubei Key Laboratory of Molecular Imaging, Wuhan, 430022 China

**Correction to: J Exp Clin Cancer Res (2018) 37: 306**

**https://doi.org/10.1186/s13046-018-0983-0**

In the publication of this article [1], there is an error in affiliation 1. The revised affiliation has now been included in this correction.

The error:

^1^ Department of Nuclear Medicine, Wuhan Union Hospital, Tongji Medical College, Huazhong University of Science and Technology, No. 1277 Jiefang Ave, Wuhan 430022, Hubei Province, China.

It should instead read:

^1^ Department of Nuclear Medicine, Union Hospital, Tongji Medical College, Huazhong University of Science and Technology, No. 1277 Jiefang Ave, Wuhan, 430022, Hubei Province, China.
